# Datamonkey 3: browser-native molecular evolution analysis

**DOI:** 10.3389/fbinf.2026.1876305

**Published:** 2026-09-09

**Authors:** Steven Weaver, Ben Murrell, Anton Nekrutenko, Sergei L. Kosakovsky Pond

**Affiliations:** 1 Institute for Genomics and Evolutionary Medicine, Temple University, Philadelphia, PA, United States; 2 Department of Microbiology, Tumor and Cell Biology, Karolinska Institutet, Stockholm, Sweden; 3 Department of Biochemistry and Molecular Biology, The Pennsylvania State University, University Park, PA, United States

**Keywords:** HyPhy, molecular evolution, phylogenetics, selection detection, WebAssembly

## Abstract

We present Datamonkey 3, a browser-native implementation of the Datamonkey web platform for molecular evolutionary analysis. By utilizing WebAssembly to execute the HyPhy analysis engine on the client side, Datamonkey 3 removes the dependency on remote computational clusters for standard analyses. This architecture ensures data sovereignty by processing sequences locally and enables an interactive workflow with immediate feedback. The platform provides a comprehensive suite of statistical methods for detecting natural selection and recombination, supported by a data curation system that identifies and corrects common formatting errors. Additionally, Datamonkey 3 incorporates data-driven runtime estimation and interactive results visualization. By shifting the computational burden to the client, Datamonkey 3 establishes a sustainable, privacy-preserving infrastructure model that scales with the user base, demonstrating the viability of client-side genomic inference. While tailored for evolutionary analysis of selection and recombination, this browser-native architecture offers a generalizable blueprint for deploying complex bioinformatic tools without server-side dependencies. Datamonkey 3 is freely available at https://v3.datamonkey.org, and all source code is available from https://github.com/veg/datamonkey3.

## 1 Introduction

Since 2005, the Datamonkey platform (Kosakovsky Pond and Frost, 2005a) has served the molecular evolution community by processing nearly one million datasets through its web interface to the HyPhy software package (Kosakovsky Pond et al., 2020). By operationalizing Maximum Likelihood methods for selection and recombination, the platform has made statistical inference accessible to researchers without local computational expertise. Although the 2018 modernization (Weaver et al., 2018) substantially re-engineered the platform by migrating from legacy Perl CGI scripts to a modern Node.js and React stack with D3 visualizations, the underlying architecture remains constrained by a centralized “upload-and-wait” model. This design imposes significant inefficiencies: even preliminary data validation requires transfer to a remote server. Consequently, users often face upload latency only to receive rejection errors for minor formatting issues. Furthermore, reliance on centralized compute introduces queue delays during high demand and compromises data sovereignty by requiring off-site transfer of genomic data.

WebAssembly (Wasm) (Haas et al., 2017), a portable binary instruction format, offers a solution to these limitations. By compiling the HyPhy engine to Wasm, we decouple analysis from server availability (Ji et al., 2024; Aboukhalil, 2024b). This approach aligns Datamonkey 3 with the “compute-to-data” model seen in tools like ViralWasm (Ji et al., 2024) and Kana (Lun and Kancherla, 2023). The shift to client-side execution is driven by three factors: privacy, interactivity, and scalability. Processing data locally ensures sensitive sequences remain on the client device. It also eliminates network latency, allowing for fluid, iterative exploration. Finally, utilizing user hardware allows the platform to scale without the infrastructure costs of centralized clusters. Here, we present Datamonkey 3, a browser-native implementation of the Datamonkey platform. This architecture moves computation to the data, employing the local client for both validation and analysis, while providing flexible support for offloading larger computation to back-end compute resources, such as HPC clusters. Beyond data sovereignty, this design enables immediate input validation, precise runtime estimation, and offline capability.

## 2 New approaches

### 2.1 Browser-native computation

**FIGURE 1:**
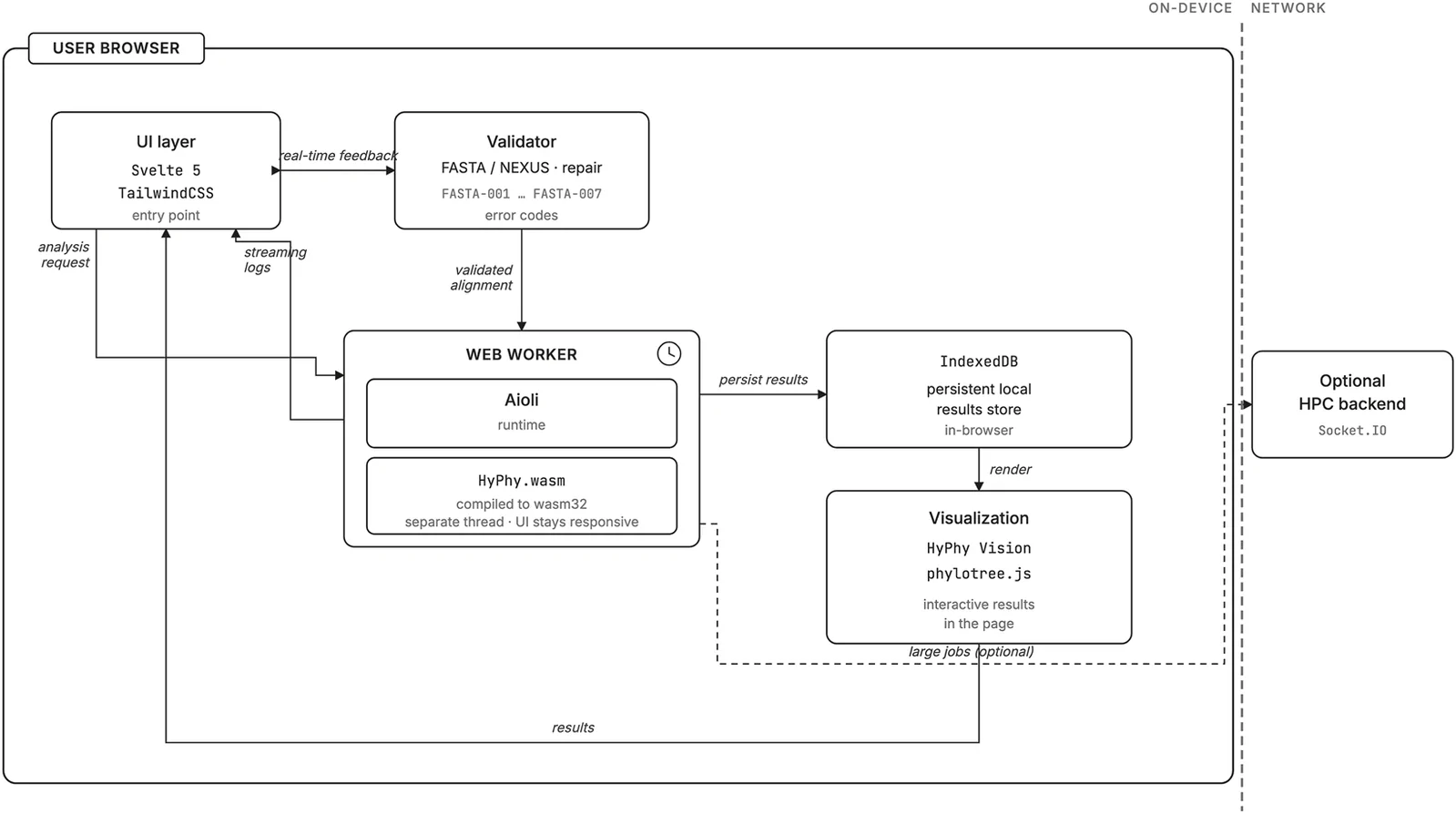
The Datamonkey 3 client-side architecture. Validation (FASTA/NEXUS, structured error codes covering malformed headers, duplicate identifiers, invalid characters, and inconsistent sequence lengths), execution (HyPhy compiled to WebAssembly via the Aioli runtime, in a dedicated Web Worker), persistent storage (IndexedDB), and interactive visualization (a HyPhy visualization component library and phylotree.js) all run inside the user’s browser. The UI layer is built with Svelte 5 (a compiler-based JavaScript framework that generates the web interface) and styled with TailwindCSS (a utility-class CSS framework for responsive layout). The clock badge on the Web Worker indicates that analyses execute asynchronously on a separate, non-UI thread, so the interface stays responsive and can stream progress while HyPhy runs. The dashed vertical line marks the device boundary. The only network connection is the dashed optional path to a backend HPC server (Socket.IO) for alignments that exceed browser memory limits.

The core innovation of Datamonkey 3 is the execution of HyPhy analyses entirely within the web browser using WebAssembly (Figure 1). We employ the Aioli library (Aboukhalil, 2024a) to interface with HyPhy compiled to Wasm, enabling the same analytical methods previously requiring server infrastructure to run on commodity hardware through any modern browser. When a user initiates an analysis, Datamonkey 3 constructs the appropriate HyPhy analysis script and executes it within a single dedicated Web Worker (WHATWG, 2024), keeping the browser interface responsive during computation. Progress updates and log messages stream to the interface in real-time. Upon completion, results are stored in the browser’s IndexedDB storage (W3C, 2018), creating a persistent local database of analyses that survives browser sessions without any server interaction.

**FIGURE 2:**
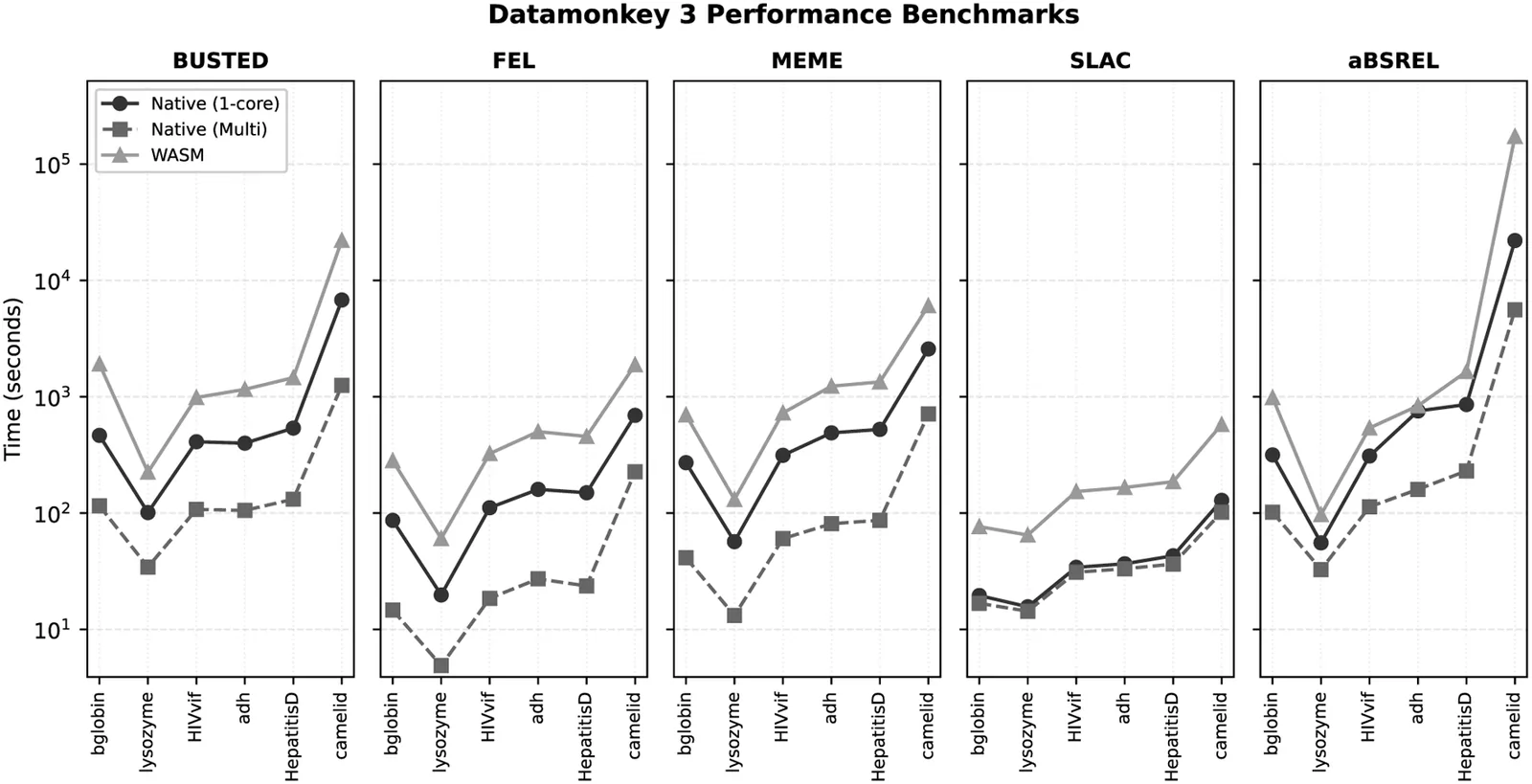
Computational performance across platforms. Execution times (log scale) for five standard selection analyses across representative datasets. Comparison includes single-core native execution (dark gray circles), multi-core native execution (medium gray squares), and browser-based WebAssembly execution (light gray triangles). WebAssembly overhead is method-dependent: modest for the likelihood-based methods (aBSREL, BUSTED, FEL, MEME, all under 2× slowdown) and substantially larger for the counting-based method SLAC (about 5× slowdown). The six representative alignments span a range of sizes and taxa: bglobin (vertebrate β -globin, 17 sequences × 144 codons), lysozyme (primate lysozyme *c*, 19 × 130), HIVvif (HIV-1 *vif*, 29 × 192), adh (*Drosophila* Adh, 23 × 254), HepatitisD (hepatitis delta virus antigen, 33 × 196), and camelid (camelid VHH antibody variable domains, 212 × 96).

To quantify the computational cost of browser-based execution, we performed two complementary benchmarks. To illustrate per-dataset behavior, we ran the five core selection methods (aBSREL, BUSTED, FEL, MEME, SLAC) on six curated representative alignments spanning a range of sizes and taxa (Figure 2). Separately, to obtain population-level overhead estimates, we ran the same methods across a larger corpus of 100 real-world alignments stratified by computational complexity (Table 2); this corpus is independent of the six representative datasets. Both native and WebAssembly benchmarks were executed on a dedicated 128-core ARM Neoverse-N1 cluster node (256 GB RAM, Rocky Linux 9.5) to ensure consistent hardware conditions. Native execution used HyPhy 2.5.93 compiled with ARM NEON SIMD optimizations, run both single-threaded (the like-for-like baseline against which we report WebAssembly overhead) and multi-threaded across all available cores (shown in Figure 2 for reference), while WebAssembly execution used HyPhy 2.5.96 compiled to wasm32 running in Chromium (headless mode). The minor version offset reflects the timing of the independent native and WebAssembly build pipelines. No algorithmic changes affecting the benchmarked methods occurred between these versions, so the offset does not influence the comparison. While WebAssembly execution incurs a computational overhead relative to native binaries (the “Native Gap”) the magnitude of the overhead varies substantially by method. Across the 100-alignment corpus, WebAssembly overhead ranged from 1.67× (67% slower for aBSREL) to 5.06× (406% slower for SLAC) relative to single-threaded native execution. The four likelihood-based methods (aBSREL, BUSTED, FEL, MEME) exhibited overheads of 1.67–1.93×, while the counting-based method SLAC showed the largest overhead at 5.06×.

Browser-based execution performance will vary depending on the user’s hardware, the analysis method, and alignment size. The platform automatically estimates runtime based on alignment characteristics and recommends server-side execution when browser-based analysis is impractical.

### 2.2 User experience

**FIGURE 3:**
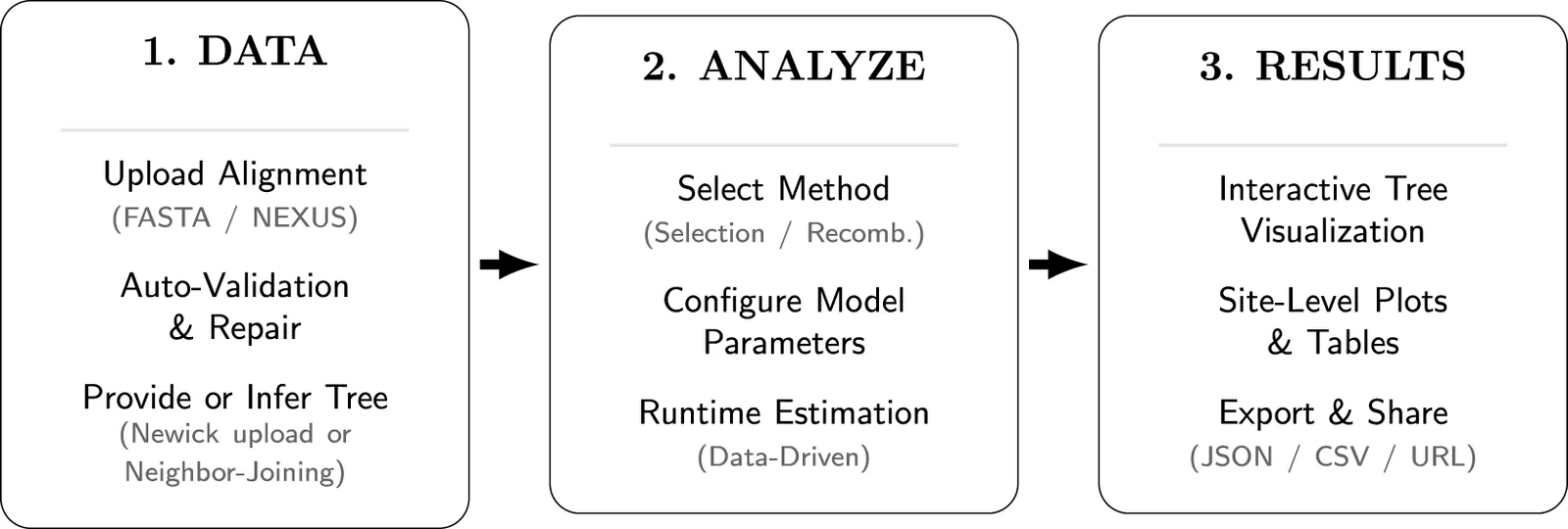
The Datamonkey 3 workflow. The interface utilizes a progressive disclosure pattern, guiding users through three distinct phases: (1) Data upload and validation, (2) Method configuration and execution, and (3) Interactive exploration of results.

Datamonkey 3 utilizes a progressive disclosure design pattern, guiding users through three distinct phases (Figure 3): (1) Data upload and validation, (2) Method configuration and execution, and (3) Interactive exploration of results. This structure reduces cognitive load by presenting options only when relevant.

**FIGURE 4:**
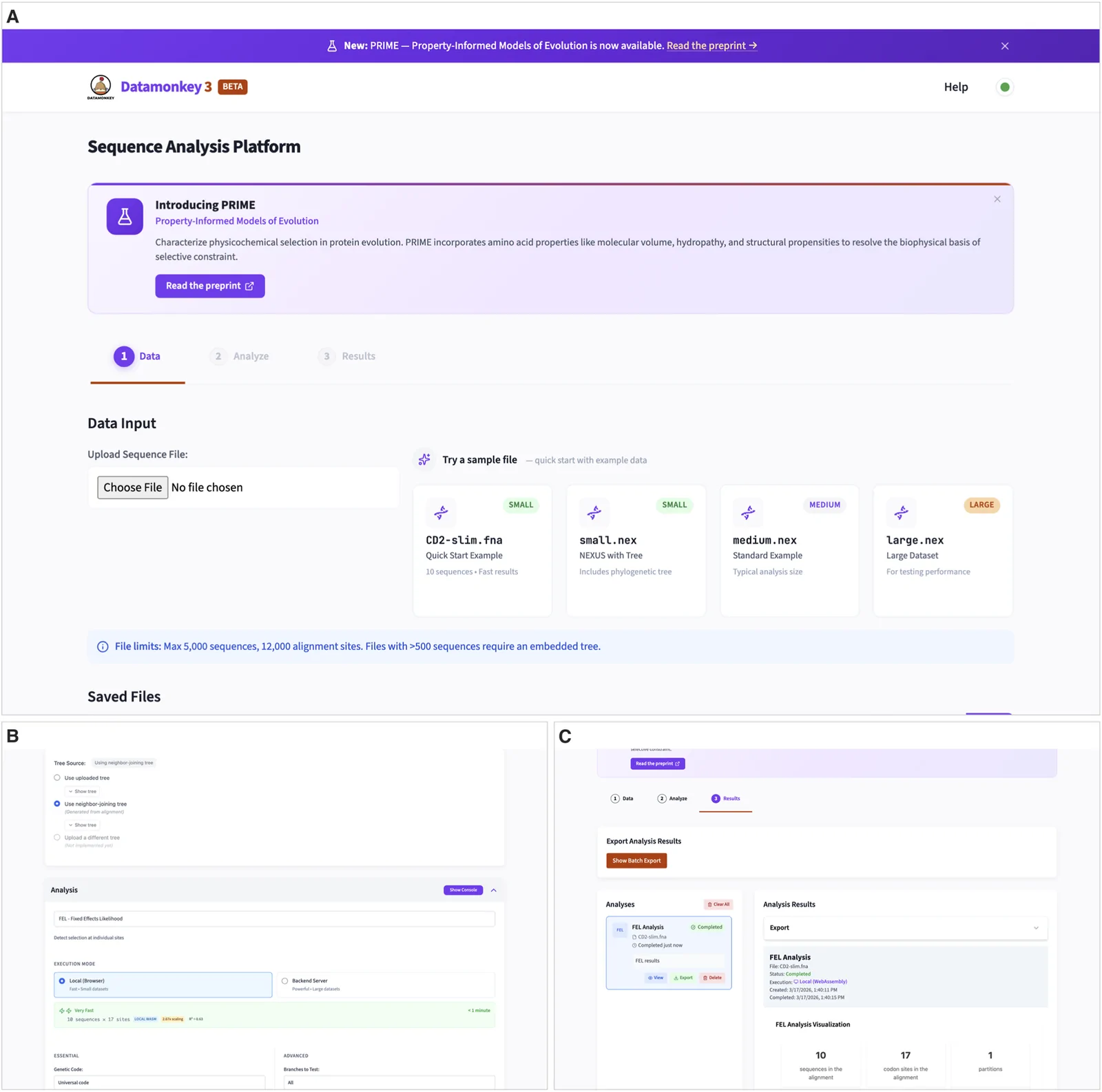
The Datamonkey 3user interface, illustrated with a FEL analysis of the CD2 alignment. **(A)** The Data tab provides sequence upload with sample datasets and displays alignment quality metrics. **(B)** The Analyze tab shows method configuration with execution mode selection (local WebAssembly or backend server) and data-driven runtime estimates. **(C)** The Results tab displays completed analyses with interactive visualizations and export options.

The Data tab (Figure 4A) handles sequence upload and validation. Users can upload FASTA or NEXUS format alignments, with intelligent validation that categorizes issues using structured error codes (FASTA-001 through FASTA-007) covering problems such as malformed or missing headers, duplicate identifiers, invalid characters, and inconsistent sequence lengths. Stop codons within coding alignments are flagged by a separate codon-alignment check. A sequence repair utility offers automated cleanup including header normalization and removal of stray whitespace and invalid characters. To ensure robust inference without manual model specification, the platform defaults to the General Time Reversible (GTR) substitution model (Tavaré, 1986) incorporating codon-level rate variation. Phylogenetic trees can be provided by the user in Newick format or inferred via neighbor-joining when no tree is supplied.

The Analyze tab (Figure 4B) presents available methods organized by analytical goal. Upon selecting a method, users configure analysis-specific options through an interface that expands to reveal advanced parameters only when requested. An interactive branch selector built on the phylotree.js library (Shank et al., 2018) enables visual specification of foreground/background partitions for methods requiring such designations. Before execution begins, data-driven runtime estimation models, calibrated against historical production analyses, provide predicted completion times based on alignment characteristics.

The Results tab (Figure 4C) maintains a history of all completed analyses with their execution timestamps and status indicators. Users can view detailed results, export data in JSON or CSV formats, batch-export multiple analyses as a ZIP archive.

### 2.3 Technical implementation

The application is built using Svelte 5, a compiler-based JavaScript framework that produces minimal, efficient code. Styling uses TailwindCSS for consistent, responsive design across devices, with accessibility features including ARIA labels and semantic HTML for screen reader compatibility. Interactive data visualizations are encapsulated within iframes, communicating with the main application via the postMessage API to ensure modularity. This architecture integrates a HyPhy visualization component library (providing reusable components for evolutionary data visualization) and native phylotree rendering to enable rich exploration of results, including annotated phylogenetic trees with interactive branch coloring, site-level selection plots, and model parameter distributions. Each analytical method includes a demonstration page with pre-loaded example datasets, providing an educational resource for users unfamiliar with specific analyses.

### 2.4 Methods available

Datamonkey 3 provides a broad collection of methods for analyzing coding sequence alignments (Table 1). These span the detection of pervasive and episodic positive selection, relaxed or intensified selection, recombination, and other evolutionary processes. Methods inherited from Datamonkey 2.0 retain their original implementations and interpretations, ensuring continuity for existing users, while new methods extend capabilities to additional evolutionary questions, including detection of convergent evolution and analysis of rate variation patterns.

**TABLE 1:** Analytical methods available in Datamonkey 3, categorized by primary application.

Method	Purpose and methodological basis
Site-level selection (Detecting specific sites under selection)	
SLAC	Counting-based detection of pervasive selection (Kosakovsky Pond and Frost, 2005b)
FEL	Maximum likelihood detection of pervasive selection (Kosakovsky Pond and Frost, 2005b)
FUBAR	Bayesian detection of pervasive selection (Murrell et al., 2013)
MEME	Detection of episodic diversifying selection (Murrell et al., 2012)
PRIME	Property-informed detection of physicochemical selection at sites (Kim et al., 2026)
Branch-level selection (Detecting specific lineages under selection)	
aBSREL	Adaptive branch-site random effects model (Smith et al., 2015)
Gene-level selection (Detecting whether a gene has undergone episodic diversifying selection)	
BUSTED	Gene-wide test for episodic selection (Murrell et al., 2015)
Comparative selection (Contrasting selective regimes)	
RELAX	Test for relaxed or intensified selection (Wertheim et al., 2015)
Contrast-FEL	Compare selection between branch groups (Kosakovsky Pond et al., 2021)
Other evolutionary analyses	
GARD	Recombination detection (Kosakovsky Pond et al., 2006)
MULTI-HIT	Multiple nucleotide substitution analysis (Lucaci et al., 2021; 2023)
BGM	Bayesian graphical model for coevolution (Poon et al., 2008)
B-STILL	Bayesian test of evolutionary stasis at invariant sites (Kosakovsky Pond et al., 2026)

### 2.5 Comparison to existing tools

Datamonkey 3 occupies a unique position in the landscape of molecular evolution analysis software. Traditional server-based web platforms like the previous Datamonkey versions and Selecton (Stern et al., 2007) require uploading data to central servers, creating privacy concerns and introducing queue delays. Similarly, comprehensive workflow management systems like Galaxy (Jalili et al., 2020) offer robust reproducibility and HPC integration but typically rely on substantial server-side infrastructure and data transfer. Conversely, desktop applications like PAML (Yang, 2007) and MEGA (Tamura et al., 2021) provide full computational control but require installation, compilation (for PAML), and local expertise. While recent browser-based tools like ViralWasm (Ji et al., 2024), Taxonium (Sanderson, 2022), and Nextstrain (Hadfield et al., 2018) demonstrate the potential of client-side computation and visualization, they typically focus on specific niche applications or surveillance workflows. However, no existing tool combines a comprehensive suite of molecular evolution analysis methods (spanning selection detection, recombination, and coevolution) with browser-native WebAssembly execution requiring zero installation. The client-side application can be deployed instantly as a static site, requiring only a modern web browser.

For researchers wishing to implement a custom backend, Datamonkey 3 can be configured to interface with any Socket.IO-compatible server capable of dispatching HyPhy jobs to local or remote HPC clusters. For users requiring computational resources beyond browser capabilities (e.g., very large alignments exceeding the 4 GB WebAssembly memory limit), the platform maintains optional connectivity to backend HPC resources. This bridge utilizes a persistent Socket.IO connection to offload analysis execution while maintaining real-time progress monitoring within the unified interface. This architecture supports session persistence, allowing users to reconnect to running jobs even after browser restarts or connectivity interruptions.

Table 2 presents a comprehensive performance comparison between browser-based WebAssembly execution and native HyPhy binaries. These benchmarks compare identical datasets that successfully completed in both environments, eliminating selection bias from differential timeout rates. The 100 alignments were stratified by computational complexity, ranging from small datasets (40 alignments with <10,000 sequence bisite products) to very large datasets (1 alignment with >500,000 sequence itsite products). The most computationally intensive datasets (13–25 per method) exceeded browser timeout thresholds and were excluded from both CLI and WASM averages to ensure valid comparison. The benchmarks demonstrate that WebAssembly overhead is highly method-dependent, ranging from 67% (aBSREL) to 406% (SLAC). Overhead predictability, measured as the ratio of interquartile range to median (IQR/median), varies substantially: SLAC shows the most consistent overhead (30% IQR/median) despite its high absolute overhead, while aBSREL, BUSTED, FEL, and MEME exhibit greater variability (44%–63% IQR/median), likely reflecting sensitivity to dataset-specific characteristics such as tree topology, sequence divergence, and optimization convergence patterns. The absolute runtimes for most datasets remain within interactive timescales (under 30 min) even for browser execution, making client-side analysis viable for typical research workflows.

**TABLE 2:** Performance comparison between browser-based WebAssemblyexecution and native HyPhy(single-threaded) on matched datasets. To eliminate selection bias, only datasets that successfully completed in both environments are included. The per-method dataset count varies because the most computationally intensive alignments exceeded browser execution limits and were excluded from both CLI and WASM averages (see §2.5, “Comparison to existing tools”). WASM overhead indicates the performance penalty relative to native execution. Benchmarks were conducted on a dedicated cluster node (128-core ARM Neoverse-N1, 256 GB RAM, Rocky Linux 9.5) using HyPhy 2.5.93 native (single-threaded with ARM NEON SIMD) and HyPhy 2.5.96 WASM (Chromium headless) to ensure consistent hardware conditions. Whereas Figure 2 reports per-dataset times for six representative alignments, this table aggregates over a separate corpus of 100 real-world alignments. Each method–dataset combination was executed once per environment on an otherwise idle node, with no separate warm-up runs; reported CLI and WASM runtimes are means across the datasets in each row, and the variability characterized in the text (IQR/median) reflects the spread of the WASM-versus-native overhead ratio across the dataset population rather than repeated-measurement variance on a single dataset.

Method	Datasets	CLI runtime(s)	WASM runtime(s)	WASMoverhead
aBSREL	75	347.2	580.1	+67.1%
BUSTED	75	624.7	1,160.1	+85.7%
FEL	84	372.1	718.9	+93.2%
MEME	77	729.6	1,261.0	+72.8%
SLAC	87	101.7	514.5	+405.9%

### 2.6 Programmatic access via Model Context Protocol

In addition to the browser interface, Datamonkey 3 exposes its full suite of analytical methods through the Model Context Protocol (MCP) (Anthropic, 2024), an open standard for connecting AI assistants and programmatic clients to external tools. The MCP server, accessible at https://mcp.datamonkey.org/mcp via Streamable HTTP transport with OAuth authentication, provides six tools: list_analyses (enumerate available methods and their parameters), validate_alignment (pre-flight checks on input data), spawn_analysis (submit a job for any supported method), get_job_status (poll job progress), get_job_results (retrieve the full HyPhy JSON output), and cancel_job. The server also provides built-in prompts for guided workflows (e.g., method selection, result interpretation) and resources describing method requirements and comparisons.

This integration enables MCP-compatible AI coding assistants to submit evolutionary analyses, monitor their progress, and interpret results within conversational workflows. For example, a researcher can instruct an AI assistant to “run FEL on my alignment to find sites under pervasive selection,” and the assistant will validate the input, submit the job, poll until completion, and summarize significant results. More complex multi-step pipelines, such as running GARD to detect recombination breakpoints followed by partition-specific FEL analyses, can be orchestrated through natural language instructions. The MCP server accepts FASTA alignments with optional Newick trees (inferring neighbor-joining trees when none are provided) and supports method-specific parameter overrides for all analytical methods. Scripted access is straightforward from any MCP-compatible client. For example, a Python client can be built against the official mcp SDK to enable batch processing and integration into computational pipelines.

### 2.7 Limitations and future directions

While WebAssembly enables powerful browser-native computation, several technical constraints should be acknowledged. The current wasm32 architecture imposes a 4 GB memory limit, restricting the size of alignments that can be processed in-browser. Based on our performance benchmarks across the same 100-alignment corpus used for Table 2 (40 small, 43 medium-small, 14 medium, 2 large, 1 very large), we established empirical thresholds for browser-based execution: alignments with sequence × site products below 50,000 complete within 30 min for most methods, while products below 200,000 remain feasible for fast methods like SLAC and FEL. In practical terms, this translates to approximately 100–150 sequences for standard gene-length alignments (500–1,000 codons) or 50–75 sequences for longer genes (2,000–3,000 codons). Memory consumption scales approximately linearly with alignment size for site-level methods (FEL, SLAC, MEME) at roughly 100–200 MB per 10,000 products, while branch-site methods (aBSREL, BUSTED) require 200–400 MB per 10,000 products due to additional model complexity. Mobile devices face stricter constraints: iOS Safari and Chrome Android typically limit WebAssembly to 300–500 MB, restricting practical analysis to alignments below 20,000 products. The platform automatically recommends server-side execution for datasets exceeding these browser capabilities.

Browser storage persistence varies across implementations, with Safari potentially auto-deleting IndexedDB data under storage pressure. Users are advised to export important results for long-term archival. Future development will focus on optimizing memory usage for larger alignments and exploring the emerging wasm64 standard (Memory64), which promises to eliminate the 4 GB barrier by allowing Wasm applications to address the full RAM of the host device. Additionally, we aim to extend analytical methods to incorporate recent advances in phylogenetic model selection and ancestral reconstruction.

## 3 Discussion

The Datamonkey 3 architecture illustrates that the historical trade-off between accessibility (web interfaces) and performance (native code) is no longer a key constraint in bioinformatics software design. By migrating the computational burden from institutional servers to the user’s edge device, the platform addresses the sustainability challenges facing public genomic resources, specifically the linear scaling of infrastructure costs with data growth. This decentralized model suggests a future where high-performance evolutionary inference functions not as a centralized service, but as a portable capability embedded within data repositories, electronic lab notebooks, and field-deployed devices. Furthermore, the portability of the underlying WebAssembly engine positions the platform’s analytical core as a potential component for emerging AI-driven workflows, enabling autonomous agents to verify evolutionary hypotheses without external API dependencies. As WebAssembly matures to support larger memory models and multi-threading, client-side execution is anticipated to become an increasingly viable alternative to centralized processing for standard bioinformatic tasks, potentially reserving institutional HPC resources for the most computationally intensive analyses. The open-source implementation of Datamonkey 3 (MIT license) supports adaptation by the research community, fostering an ecosystem where statistical methods possess the same portability as the data they analyze.

## Data Availability

Datamonkey 3 is freely available at https://v3.datamonkey.org. Client-side source code is available under the MIT license at https://github.com/veg/datamonkey3. Documentation and tutorials can be accessed at http://help.datamonkey.org. A reference Socket.IO-compatible backend server, suitable for connecting to local or remote HPC clusters, is available at https://github.com/veg/datamonkey-js-server. HyPhy source code and WebAssembly compilation instructions are available at https://github.com/veg/hyphy (Kosakovsky Pond et al., 2020).
